# Changes of Hand Switching Costs during Bimanual Sequential Learning

**DOI:** 10.1371/journal.pone.0045857

**Published:** 2012-09-21

**Authors:** Sabrina Trapp, Jöran Lepsien, Bernhard Sehm, Arno Villringer, Patrick Ragert

**Affiliations:** 1 Max Planck Institute for Human Cognitive and Brain Sciences, Leipzig, Germany; 2 Mind and Brain Institute, Charité and Humboldt University, Berlin, Germany; Bielefeld University, Germany

## Abstract

Many tasks in our daily life demand not only the use of different fingers of one hand in a serial fashion, but also to alternate from one hand to the other. Here, we investigated performance in a bimanual serial reaction time task (SRTT) with particular emphasis on learning-related changes in reaction time (RT) for consecutive button presses for homologous index- and middle fingers. The bimanual SRTT consisted of sequential button presses either with the left or right index- and middle-finger to a series of visual letters displayed on a computer screen. Each letter was assigned a specific button press with one of four fingers. Two outcome measures were investigated: (a) global sequence learning as defined by the time needed to complete a 15-letter SRTT sequence and (b) changes in hand switch costs across learning. We found that bimanual SRTT resulted in a global decrease in RT during the time course of learning that persisted for at least two weeks. Furthermore, RT to a button press showed an increase when the previous button press was associated with another hand as opposed to the same hand. This increase in RT was defined as switch costs. Hand switch costs significantly decreased during the time course of learning, and remained stable over a time of approximately two weeks. This study provides evidence for modulations of switch costs during bimanual sequence learning, a finding that might have important implications for theories of bimanual coordination and learning.

## Background

Many tasks in our daily life demand not only to use different fingers of one hand in a serial fashion, but also alternate from one hand to the other. Thus, knowledge about errors and effects typically found in bimanual performance and understanding their respective mechanisms not only contributes to theorizing in human motor control, but has major implications for practical applications, e.g. in robotics and clinical settings. In the past, numerous studies have examined the mechanisms underlying unilateral motor skill learning using a serial reaction time task (SRTT), typically revealing a learning effect after several repetitions when compared to a random sequence (for review see [1]). Studies probing for neural correlates of the production of sequential movements identified key areas such as motor-related cortical areas including the primary motor cortex [2], [3], prefrontal areas [4], [5], the cerebellum [6] and occasionally, the basal ganglia [7]. It has also been suggested that interhemispheric inhibition (IHI) is involved in simple and complex sequences of finger movements by suppressing the activity of the contralateral hemisphere [8]–[10]. A major focus in learning- related studies assessing performance changes during SRTT is whether skills acquired at one hand would be transferred to the other hand [11], [12]. Here, IHI between M1 cortices is known to play an important role for intermanual transfer [13]. Apart from the considerable knowledge regarding unilateral motor sequence learning, surprisingly little is known about the neurophysiological mechanisms of bilateral engagement of hands in a sequential manner. This however is an important issue in daily life situations where we use our two hands together such as in typing on a keyboard or playing a piano. Early experimental work provided inconsistent effects for reaction times (RTs) that are associated with switching between fingers of the same hand and between hands [14]–[17] a phenomenon that certainly requires further investigation in future studies. In the present study, we were interested in learning effects of a bimanual SRTT with particular emphasis on modulations in RTs associated with switches between hands. We expected an increase in RT when the button press is associated with a switch between hands. There is evidence that motor execution is hierarchically controlled and follows a tree-traversal process [18]. Here, performance in a SRTT depends on the number of nodes that have to be traversed. According to this model, a transition between fingers from different hands would require at least one more node to be traversed than a transition between fingers from one hand. This is assumed to result in an increase in response latencies. Therefore, we expected higher increases in RT when two subsequent button presses are associated with two hands. It is however important to note that the data supporting this model were derived from subjects performing sequential button presses according to a previously learnt and memorized sequence. In contrast, the present study investigates performance to visually presented stimuli.

In addition, we expected that these switch costs between hands will progressively *decrease* during the time course of motor skill learning. This hypothesis was motivated by the fact that previous studies consistently indicated that learning is associated with task-specific functional alterations in motor-related areas, a finding that seems to be associated with an optimization of processing resources within and between hemispheres sub-serving different stages of motor skill learning (for review see [19]). Based on these findings, we reasoned that this might translate into a decrease in hand switch costs over time. Finally, we aimed to investigate the long-term retention of the learning-related decrease in switch costs by re-testing the subjects two weeks later under the same experimental paradigm.

## Materials and Methods

### Participants

Twenty neurologically healthy subjects (mean age = 25.22 years, SD = 3.62, 12 females) gave written informed consent to participate in the experiment according to the declaration of Helsinki and the ethics committee of the University of Leipzig approved the study. All subjects were right- handed as assessed by the Edinburgh handedness scale [20]. The volunteers were recruited from the Max Planck Institute for Human Cognitive and Brain Sciences and were financially compensated for their participation. All participants had normal or corrected to normal vision.

### Study Design

**Figure 1:**
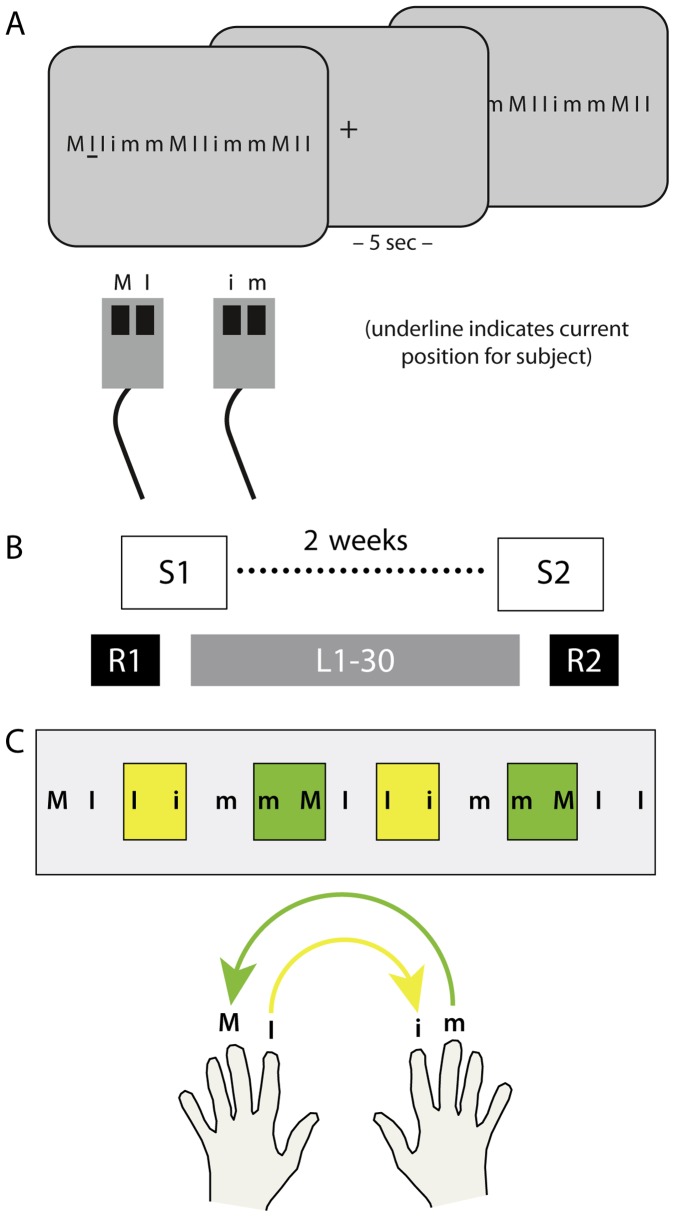
Experimental setup and design of the SRTT. (**A**) A learning sequence (MIIimmMIIimmMII) was displayed on a computer screen and participants were asked to respond as fast and accurate as possible once they perceived the sequence by pressing the corresponding button on the response button devices. Feedback regarding average RT and number of errors was given by the end of each sequence (not shown). The inter-stimulus interval between each sequence presentations was 5000 ms in order to avoid muscle fatigue during the experimental procedure. (**B**) During the SRTT, participants performed the learning sequence 30 times (L1–30). Additionally, a random sequence (ImmiMMIiMMimmMM) was also presented before and after L1–30 (R1 and R2 respectively). (**C**) The learning sequence consisted of a total number of 5 within hand switches (between index and middle fingers) and 4 between hand switches of homologous fingers (yellow: left to right index finger; green: right to left middle finger). For details see text. (M = left middle finger, I = left index finger, i = right index finger, m = right middle finger).

In the present study we used a SRTT. The SRTT consisted of sequential finger presses to a series of visual letters displayed on a computer screen. Stimulus presentation and behavioural response collection were controlled by Presentation software (Neurobehavioral Systems, Inc., version 14.7). Participants were seated in front of the computer screen with left and right index and middle finger placed on four corresponding response-buttons (see Fig. 1). The distance to the computer screen was 90 cm. The stimuli consisted of a set of four different letters (M, I, m and i), whereas each letter corresponded to a predefined response button (uppercase letters indicated the left hand, M = left middle finger, I = left index finger, and lowercase letters indicated the right hand, m = right middle finger, i = right index finger). The learning sequence consisted of 15 letters (M I I i m m M I I I m m M I I). Prior to the main experiment, subjects performed a single familiarization session consisting of three trial repetitions using the following sequence: M M M I I I I I I m m m M I i. The experiment started with the presentation of a sequence consisting of 15 letters, which included four between-hand transitions (two switches from left to right index finger and two switches from right to left middle finger) and five within-hand transitions (three switches from left middle to index finger and two switches from right index to middle finger). The letters were presented centrally in black font (height = 1.5 cm on screen) on a light grey background. Additionally, a random sequence (I m m i M M I i M M i m m M M) was presented before and after the learning sequence, which also contained 15 letters and included five hand switches. Participants were asked to respond as fast and accurate as possible once they perceived the sequence by pressing the corresponding button on the response button devices. The task was self-paced and had no time limitation to respond. A black line underneath the respective letter position served as visual cue indicating which button to press. Feedback regarding average RTs and number of errors was given by the end of each sequence. The inter-stimulus interval between each sequence presentations was 5000 ms in order to avoid muscle fatigue during the experimental procedure. During the SRTT, participants performed the training sequence 30 times. All participants were explicitly informed about the amount of sequences and that the goal of the experiment was to investigate motor learning. The experiment included a recovery measurement under the same experimental procedures as described above after approximately 2 weeks (stability measurement). Task and experimental set-up are illustrated in Fig. 1.

### Behavioural Measurements

**Figure 2:**
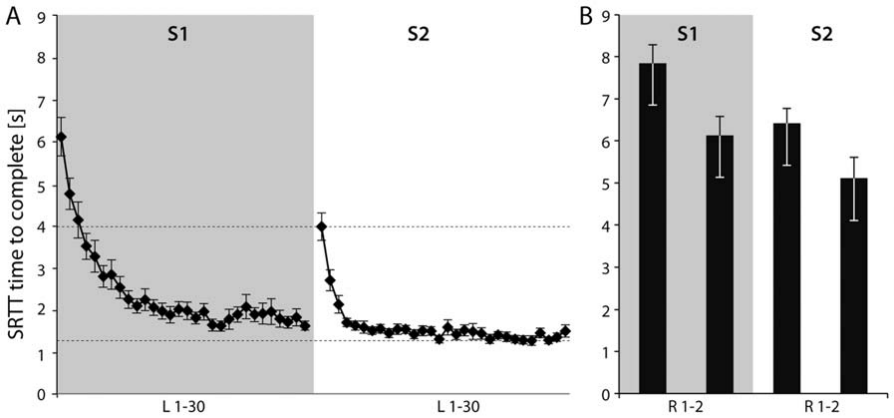
Global SRTT performance. SRTT performance (time to complete [s]) for the learning sequence (**A**) and the random sequence (**B**) in session 1 (S1) and two weeks later (S2). X-axis: L 1–30 indicates the number of repetitions for the learning sequence (L). R 1–2 indicates the number of repetitions for the random sequence (R). Dashed lines indicate performance in the SRTT in the first and last learning sequence of S2. Please note that there was a significant improvement over time for S1 and S2 for the learning and random sequence. However, the improvement was significantly more pronounces for the learning sequence tested.

First, in order to probe for global learning effects in the SRTT, we analysed the effect of time (30 repetitions, expressed as the total time to complete the sequence (calculated by adding all correct mean RTs for each of the 15 letters)) for the learning sequence (Fig. 2). Data were averaged across our group of 20 subjects. Additionally, we compared RT differences between session 1 and session 2 (S1, S2) in order to probe for stability effects (Fig. 2).

**Figure 3:**
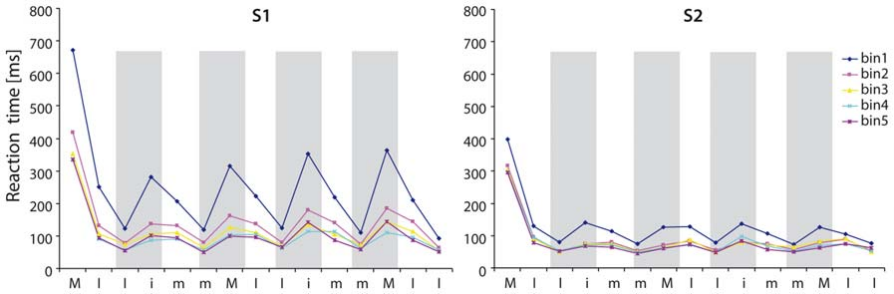
RT [ms] for each button press for the learning sequence. X-axis represents the button press in the sequence (M = left middle finger, I = left index finger, i = right index finger, m = right middle finger). We split the 30 repetitions of the learning sequence (L1–30) into five time bins (bin 1–5), consisting of 6 learning sequences each, averaged across subjects and across button presses. The primary goal using this approach was to potentially differentiate between early and late SRTT learning including the development of switch costs over time (within S1 and S2). Positions where switch costs occur within the learning sequence are illustrated by grey bars. Please note that there was a significant reduction in hand switch costs during the time course of learning (S1). For details see text. (M = left middle finger, I = left index finger, i = right index finger, m = right middle finger).

Second, we intended to investigate the time course of SRTT learning separately for each letter (15 letters per sequence), in order to reveal learning effects related to pressing buttons from same or different hands, respectively. To this end, we split the 30 repetitions of the learning sequence into five time bins, consisting of 6 sequences each, averaged across subjects and across button presses. The primary goal using this approach was to potentially differentiate between early and late SRTT learning including the development of switch costs over time (see also Fig. 3). We only considered correct mean RT for this analysis, i.e. wrong button presses were not included in the analysis. Error rates were very low (<0.5%) and were thus not taken as dependent variables in the statistical analysis. For computing hand switch costs, we calculated – for each of 5 bins – the mean percentage difference (increase/decrease) in RT between two subsequent button presses that are associated with two hands. We used the following formula: 100−(mean RT _before switch_/mean RT _after switch_)*100. The computation of percentage differences instead of subtracting RTs was particularly important for our experiment since it was a learning task where RTs decrease over the time course of learning.

### Statistical Analysis

In order to test the influence of sequence repetition and the stability of learning effects on total RT, a 2×30 repeated-measures analysis of variance (ANOVA_RM_) tested the influence of sequence repetition (30 trials) and stability (S1, S2) upon total RT per sequence.

For examining learning effects to each letter of the sequence, a 2×5×15 ANOVA_RM_ tested the influence of stability (S1, S2), sequence repetition (30 sequences, split into 5 time bins), and letter position (15 letters in the sequence) upon mean RT per letter.

For analyzing the switch costs, a 2×5×4 ANOVA_RM_ tested the influence of stability (S1, S2), sequence repetition (30 trials, split into 5 time bins), and position of hand switch in the sequence (1^st^, 2^nd^, 3^rd^ or 4^th^ hand switch) upon percentage gain in mean RT for hand switches. For the hand transitions, the between hand switch positions included two switches from left to right (letter I to i, 1^st^ and 3^rd^ position) and two switches from right to left hand (letter m to M, 2^nd^ and 4^th^ position).

## Results

### Global SRTT Improvement

Performing the bimanual SRTT in S1 resulted in a significant decrease in RT during the learning sequence from 6.11±2.08 s (1^st^ sequence, mean ± stdev.) to 1.62±0.48 s (last sequence, see also Fig. 2A). This pattern was statistically supported by an ANOVA_RM_ with factor sequence repetition [F(29,551) = 54.73, p<0.001]. Subjects were faster during the second measurement (S2), as revealed by a main effect of stability [F(1,19) = 18.12, p<0.001]. Finally, the interaction [sequence repetition × stability] reached significance [F(29,551) = 8.57, p<0.001]. A visual inspection of the learning curves suggests that the interaction was driven by a steeper drop off after approximately 5 sequence repetitions in S2 (see Fig. 2A). However, subjects still performed significantly slower at the beginning of S2 as compared to the last sequence of S 1 (p<0.001, Bonferroni-corrected t-test, see also Fig. 2A), rendering any consolidation effects unlikely. While the SRTT performance in the random sequences was also significantly reduced from 7.86±1.96 s (1st sequence) to 6.13±2.03 s (last sequence, p<0.001, Bonferroni-corrected t-test, see Fig. 2B), the improvement for the random sequences (1.73±1.78 s 1st random vs. last random sequence) was significantly less pronounced as compared to the learning sequences (4.49±1.87 s 1^st^ learning vs. last learning sequence, p<0.001, Bonferroni-corrected t-test).

### SRTT Learning on Single-trial Level

Figure 3 visualizes the mean RT for each letter in the sequence. Each curve is an average over 6 sequences each (5 time bins) for all subjects tested. As can be seen, mean RT consistently increased when subjects had to switch from left to right index-finger (Ii) or from right to left middle-finger (mM), respectively (switch costs, transparent grey background in Fig. 3). During the time course of learning, RT decreased significantly with a strong improvement in performance between the first and second time bin (sequence 1–10). Figure 3 illustrates switch costs both for S1 and S2, grouped into 5 time bins each. Switch costs for the first five trials in S2 are already approximately at the same level as in the middle of S1 (trials 13–18, 3rd bin).

To support these pattern statistically, we conducted an ANOVA_RM_ which revealed a significant effect of sequence repetition [F(4,76) = 92.32, *p*<0.001]. Furthermore, subjects were faster during the second measurement, as demonstrated by a main effect of stability [F(1,19) = 31.5, *p*<0.001]. RTs at the beginning of S2 started on a much lower scale, comparable to the RTs at the middle of the first measurement, supported by a significant interaction [sequence repetition × stability] [F(4,76) = 30.91, *p*<0.001]. There was also a main effect of letter position in the sequence [F(14,266) = 114.28, *p*<0.001]. The letter position interacted with sequence repetition [F(56,1064) = 11.68, *p*<0.001] and stability [F(14,266) = 9.73, *p*<0.001]. As a matter of fact, the three-way interaction between letter position, sequence repetition and stability was significant as well [F(56,1064) = 6.06, *p*<0.001].

In order to examine the above mentioned significant effects of letter position in terms of switch costs, the percentage gain in mean RT was used as a dependent variable in an ANOVA_RM_. Here, a significant reduction of hand switch costs over time could be observed by a main effect of sequence repetition [F(4,76) = 3.91, *p* = 0.022]. The position of the hand switch in the sequence was not significant [F(3,57) = 1.25, p = 0.239]. Thus, no significant differences for a switch from left to right hand (1^st^ and 3^rd^ position) and right to left hand (2^nd^ to 4^th^ position) can be identified in this data set. Additionally, there were significant differences for switch costs between S1 and S 2, with generally lower switch costs during S2 [F(1,19) = 6.18, *p* = 0.022].

## Discussion

In the present study, subjects had to learn a bimanual SRTT on two separate days including button presses with their right and left index- and middle finger. On the first day, bimanual SRTT learning resulted in a significant reduction in the total time needed to complete the sequence. This effect was significantly different from the performance in a random sequence, indicating sequence-specific learning related SRTT improvements. Interestingly, re-testing the subjects approximately 2 weeks later with the same experimental paradigm revealed a long-lasting learning effect in SRTT performance. Furthermore, we showed that RTs for button presses were significantly slower when subjects had to alternate their response between hands. These hand switch costs decreased during the time course of learning. The reduction of hand switch costs occurred very rapidly during the first five repetitions of the learning sequence and reached an asymptote after approximately 10 repetitions. The SRTT learning effect persisted at least over a period of two weeks as demonstrated by an additional stability measurement. The decrease of switch costs, i.e., the learning effects after the initial trials, was much more accentuated after two weeks. To our knowledge, this is the first study that examines learning and stability effects in mean RT costs for between hand transitions.

Previous studies have reported rather inconsistent results for RTs within and between hands. For example, some authors found that the time between successive keystrokes were shorter when conducted with different hands as opposed to same hands [16], [21], [22]. In contrast, other studies reported faster within-hand transitions [15], [17]. However, there were several differences in design and task between these studies. Most importantly, in some of these studies, subjects were precued for one of multiple responses. Rosenbaum and Kornblum suggested that the discrepancies between these or related studies are due to different response preparation characteristics [14]. When various responses are possible and are – presumably – simultaneously prepared, the time to switch within one hand is longer. The authors speculated that in this particular case, subjects have to choose between alternative movement representations, and this is assumed to be more difficult with high feature similarity (i.e., two fingers of one hand have more movement features in common as opposed to two fingers from different hands). In our study, each response was specified by a visually presented letter on screen and thus strongly suggested to prepare each button press individually. In this case, Rosenbaum and Kornblum (1983) suggested that in order to perform several movements, the movement representation used for the *previous* executed movement is modified for the execution of the *subsequent* movement. Here, the modification process depends on the number of features that have to be modified. As a matter of fact, there are more features to be modified when the subsequent execution is done with a different hand. However, there are several differences between our study and previous studies, e.g., the task was self-paced and all stimuli were presented visually while performing a single sequence repetition. Hence, we believe that a direct comparison between previous studies has to be taken with caution. It is furthermore important to keep in mind that the level of explanation in the above mentioned studies is mainly based on cognitive concepts without taking into account the underlying neurophysiological mechanisms. However, it is reasonable to assume that at least in the early learning stage, there might be a response conflict between fingers of both hands that is related to interhemispheric rivalry and/or inhibition (IHI) between both motor cortices M1 [23], [24]. Therefore, hand switch costs during the initial stage of bimanual SRTT learning might at least be partially explained by a predetermined interhemispheric inhibition between both M1. In fact, interhemispheric inhibition has been repeatedly demonstrated under resting and task conditions [25]. Alternatively, mutual inhibition between premotor cortices (PMC) and/or between PMC and contralateral M1 [26], [27] might also be potential candidate mechanisms. Apart from “baseline” transcallosal inhibition between both M1, learning-related changes in IHI have also been observed in subjects performing a unimanual sequential pinch force task [28]. Camus and colleagues (2009) demonstrated that motor learning was associated with a significant reduction in IHI from the dominant (trained) to the non-dominant (untrained) M1. Based on these findings, it might be an interesting hypothesis for future studies to test whether or not the observed reduction in switch costs during SRTT learning might be related to a modulation of IHI between both M1.

In summary, our behavioral results provide evidence that (a) RTs in a bimanual SRT task are significantly slower when switching *between* hands and (b) that these hand switch costs can be reduced through learning. Our study design leaves some issues that need to be addressed more thoroughly in future studies. For example, in our learning sequence, switching between hands occurred only between index fingers (from left to right) and middle fingers (from right to left) in one direction. Therefore, we cannot give a detailed view about the effects of directionality on hand switch cost. Furthermore, there is evidence that subjects are faster when performing a task with two hand homologous fingers as compared to two hand non-homologous finger combinations [29]. Since in this study, we only investigated switch costs between homologous fingers, we cannot make inferences about learning effects of hand switches for non-homologous fingers. Moreover, the underlying neuronal mechanisms remain elusive at this stage and certainly require further investigation. Potential determinants for learning-related alterations in hand switch costs could be cognitive in nature, such as improved response certainty by over-learnt stimulus response mappings. Furthermore, learning could be determined by neurophysiological changes in interhemispheric inhibition between primary motor cortices. The question whether just one or more factors contribute to bimanual SRTT learning and associated reductions in hand switch costs is beyond the scope of the present study and has to be addressed in future experiments, possibly with the help of interventional studies using non-invasive brain stimulation.
